# On Entropic Framework Based on Standard and Fractional Phonon Boltzmann Transport Equations

**DOI:** 10.3390/e21020204

**Published:** 2019-02-21

**Authors:** Shu-Nan Li, Bing-Yang Cao

**Affiliations:** Key Laboratory for Thermal Science and Power Engineering of Ministry of Education, Department of Engineering Mechanics, Tsinghua University, Beijing 100084, China

**Keywords:** entropy density, entropy flux, entropy production rate, classical irreversible thermodynamics (CIT), extended irreversible thermodynamics (EIT), phonon heat transport, Boltzmann transport equation (BTE)

## Abstract

Generalized expressions of the entropy and related concepts in non-Fourier heat conduction have attracted increasing attention in recent years. Based on standard and fractional phonon Boltzmann transport equations (BTEs), we study entropic functionals including entropy density, entropy flux and entropy production rate. Using the relaxation time approximation and power series expansion, macroscopic approximations are derived for these entropic concepts. For the standard BTE, our results can recover the entropic frameworks of classical irreversible thermodynamics (CIT) and extended irreversible thermodynamics (EIT) as if there exists a well-defined effective thermal conductivity. For the fractional BTEs corresponding to the generalized Cattaneo equation (GCE) class, the entropy flux and entropy production rate will deviate from the forms in CIT and EIT. In these cases, the entropy flux and entropy production rate will contain fractional-order operators, which reflect memory effects.

## 1. Introduction

Entropic and thermodynamic frameworks in heat transport have attracted increasing attention in recent years [1,2,3]. In equilibrium thermodynamics, the Clausius statement restricts the direction of heat transfer, which guarantees the non-negativity of entropy generation. Further descriptions such as the entropy production rate require irreversible and non-equilibrium thermodynamics. In classical irreversible thermodynamics (CIT) [3,4,5], the entropy density $s_{C}=s_{C}\left(x,t\right)$ is written as $s_{C}=\int T^{-1}cdT$ with T=T(x,t) the local temperature and c the specific volumetric heat capacity, while the entropy flux $J_{C}=J_{C}\left(x,t\right)$ is $J_{C}=T^{-1}q$ with q=q(x,t) denoting the heat flux. Here, the subscript C means that the entropy and entropy flux are expressed in the framework of CIT. According to the entropy balance equation, the CIT entropy production rate $\sigma_{C}=\sigma_{C}\left(x,t\right)$ is obtained as

(1) $$\sigma_{C}=\partial_{t}s_{C}+\nabla \cdot J_{C}=q\cdot \nabla \left(\frac{1}{T}\right)$$

For classical Fourier’s law, q+λ∇T=0 with λ the thermal conductivity, $\sigma_{C}$ is non-negative as if the thermal conductivity is positive. However, Fourier’s law indicates infinite speeds of heat propagation for constant material properties. Non-Fourier heat conduction models therefore arise [6,7,8,9,10,11,12,13]. The Cattaneo-Vernotte (CV) model [8,9] is “the most obvious and simple generalization of Fourier’s law that will give rise to finite speeds of propagation” [6]: $q+\tau \partial_{t}q=-\lambda \nabla T$ with τ standing for the relaxation time. The CV model reflects relaxation in heat conduction, while there are other non-Fourier effects such as nonlocality and nonlinearity. Nonlocal heat conduction is widely observed in phonon heat transport, i.e., the phonon hydrodynamic and Guyer-Krumhansl (GK) models [11]. Typical examples for nonlinear heat conduction are the Lagrange multiplier [14], tempered diffusion [15] and thermomass [16,17] models. These nonlinear models predict an interesting phenomenon termed flux-limited behavior [18], namely that the heat flux tends to a finite upper bound as the temperature gradient increases. The flux-limited behavior is sometimes paired with size effects that the macroscopic expressions will not exist for small-scale heat conduction problems [19].

One inconsistency caused by some of these models is the negativity of $\sigma_{C}$ [20,21]. In order to overcome this inconsistency, generalized formulations for the entropic functionals were developed. A commonly used approach is to introduce non-equilibrium macroscopic quantities such as q into the entropy density. For instance, in extended irreversible thermodynamics (EIT) [3], the entropy density is defined by $s_{E}=s_{C}-\frac{\tau }{2\lambda T^{2}}q\cdot q$, which enables the CV model to satisfy a non-negative entropy production. The subscript E means that the entropic framework is defined in EIT. Because the entropy production rate depends on not only the entropy density but also the entropy flux, investigations on the generalized entropy flux are likewise necessary. The nonlocal entropy flux J commonly takes a form as $J=J_{C}+K$ [1], wherein K is the so-called entropy-density extra flux. Although these entropic functionals were well-discussed for macroscopic heat conduction models and proved by Grad’s method in the kinetic theory of gases, the entropic framework was not studied as much for phonon heat transport based on the phonon Boltzmann transport equation (BTE). Moreover, the factional-order heat conduction models and their corresponding factional BTEs have not been investigated.

In the present work, we mainly study the macroscopic estimations for the entropy density and entropy flux based on the phonon BTE with the relaxation time approximation [22]:

(2) $$\partial_{t}f+v_{g}\cdot \nabla f=\frac{f_{0}-f}{\tau }$$

wherein $v_{g}$ is the phonon group velocity, f=f(x,t,k) is the distribution function, k denotes the wave vector, $f_{0}=\frac{1}{\exp\left(\hbar \omega /k_{B}T\right)-1}$ is the equilibrium distribution, ℏ is the reduced Planck constant, ω is the angular frequency, and $k_{B}$ is the Boltzmann constant. We consider the entropic concepts in Boltzmann-Gibbs (BG) statistical mechanics. Using the power series expansion, the entropic concepts in statistical mechanics are approximated by macroscopic quantities. Besides Equation (2), several fractional BTEs are also discussed, which educes a class of fractional-order heat conduction models termed generalized Cattaneo equation (GCE) [23]. 

## 2. Standard Boltzmann Transport Equation

We establish macroscopic quantities including the phonon energy density e=e(x,t), heat flux q and flux of heat flux Q=Q(x,t):

(3) $$e=\int f\hbar \omega dk,\;q=\int v_{g}f\hbar \omega dk,\;Q=\int v_{g}v_{g}f\hbar \omega dk$$

The conventional temperature is defined in the sense of equilibrium or local equilibrium. Here, we use Chen’s definition of the local temperature in non-Fourier heat conduction [22], which is a measure of the local energy density, namely, de=cdT. Upon multiplying Equation (2) by ℏω and integrating it over the wave vector space, we obtain the local energy conservation equation $\partial_{t}e=-\nabla \cdot q$. Similarly, multiplying Equation (2) by $v_{g}\hbar \omega$ and integrating yields $q+\tau \partial_{t}q=-\tau \nabla \cdot Q$. With $\lambda =\frac{1}{3}{\left|v_{g}\right|}^{2}c\tau$, the CV model is immediately recovered. 

The BG entropy density for the phonon distribution function $s_{f}=s_{f}\left(x,t\right)$ is written as

(4a) $$s_{f}=k_{B}\int \left[\left(f+1\right)\ln\left(f+1\right)-f\ln f\right]dk$$

whose time derivative reads

(4b) $$\partial_{t}s_{f}=k_{B}\int \partial_{t}f\left[\ln\left(f+1\right)-\ln f\right]dk$$

The subscript *f* means that the entropic framework is defined in terms of the distribution function and statistical mechanics. Upon substituting Equation (2) into Equation (4b), we arrive at

(5) $$\partial_{t}s_{f}=-\nabla \cdot \left\{\int v_{g}k_{B}\left[\left(f+1\right)\ln\left(f+1\right)-f\ln f\right]dk\right\}+k_{B}\int \frac{f_{0}-f}{\tau }\ln\frac{f+1}{f}dk$$

From Equation (5), we can find that the BG entropy flux $J_{f}=J_{f}\left(x,t\right)$ takes the following form

(6) $$J_{f}=\int v_{g}k_{B}\left[\left(f+1\right)\ln\left(f+1\right)-f\ln f\right]dk$$

while the BG entropy production rate $\sigma_{f}=\sigma_{f}\left(x,t\right)$ is given by

(7) $$\sigma_{f}=k_{B}\int \frac{f_{0}-f}{\tau }\ln\frac{f+1}{f}dk$$

One can acquire $\partial_{T}s_{f=f_{0}}=k_{B}\int \partial_{T}f_{0}\ln\frac{f_{0}+1}{f_{0}}dk$ and noting that $\frac{\hbar \omega }{k_{B}T}=\ln\frac{f_{0}+1}{f_{0}}$, $\partial_{T}s_{f=f_{0}}=T^{-1}\int \partial_{T}f_{0}\hbar \omega dk=T^{-1}c$. Thus, we have $s_{f=f_{0}}\equiv s_{C}$ and when $f\neq f_{0}$ yet $\left|f_{0}-f\right|\ll f_{0}$, $s_{f}$ can be expanded as

(8) $$s_{f}=s_{f=f_{0}}+k_{B}\int \left(f-f_{0}\right)\ln\frac{f_{0}+1}{f_{0}}dk+k_{B}\int -\frac{{\left(f-f_{0}\right)}^{2}}{2f_{0}\left(f_{0}+1\right)}dk+k_{B}\int O{\left(f-f_{0}\right)}^{3}dk$$

In Equation (8), $s_{f=f_{0}}\equiv s_{C}$ is the zero-order term, and substituting $\frac{\hbar \omega }{k_{B}T}=\ln\frac{f_{0}+1}{f_{0}}$ into the second term in Equation (8) yields

(9) $$k_{B}\int \left(f-f_{0}\right)\frac{\hbar \omega }{k_{B}T}dk=\frac{1}{T}\int \left(f-f_{0}\right)\hbar \omega dk=0$$

Through combining with Equation (2), the third term in Equation (8) becomes

(10) $$k_{B}\int -\frac{{\left(f-f_{0}\right)}^{2}}{2f_{0}\left(f_{0}+1\right)}dk=\frac{k_{B}\tau }{2}\int \frac{\left(f-f_{0}\right)}{f_{0}\left(f_{0}+1\right)}\partial_{t}fdk+\frac{k_{B}\tau }{2}\int \frac{\left(f-f_{0}\right)v_{g}\cdot \nabla f}{f_{0}\left(f_{0}+1\right)}dk$$

In the right-hand side of Equation (10), the first term can be simplified as follows

(11) $$\begin{array}{l} \frac{k_{B}\tau }{2}\int \frac{\left(f-f_{0}\right)}{f_{0}\left(f_{0}+1\right)}\partial_{t}fdk=\frac{k_{B}\tau }{2}\left[\int \frac{\left(f-f_{0}\right)}{f_{0}\left(f_{0}+1\right)}\partial_{t}f_{0}dk+\int \frac{\left(f-f_{0}\right)}{f_{0}\left(f_{0}+1\right)}\partial_{t}\left(f-f_{0}\right)dk\right]\\ =-\frac{\tau }{2}\partial_{t}\left(\frac{1}{T}\right)\int \left(f-f_{0}\right)\hbar \omega dk+\frac{k_{B}\tau }{2}\int O\left[\partial_{t}{\left(f-f_{0}\right)}^{2}\right]dk\\ =\frac{k_{B}\tau }{2}\int O\left[\partial_{t}{\left(f-f_{0}\right)}^{2}\right]dk \end{array}$$

while the second term in the right-hand side of Equation (10) is rewritten as

(12) $$\begin{array}{l} \frac{k_{B}\tau }{2}\int \frac{\left(f-f_{0}\right)v_{g}\cdot \nabla f}{f_{0}\left(f_{0}+1\right)}dk=\frac{k_{B}\tau }{2}\int \frac{\left(f-f_{0}\right)v_{g}\cdot \left[\nabla f_{0}+\nabla \left(f-f_{0}\right)\right]}{f_{0}\left(f_{0}+1\right)}dk\\ =\frac{k_{B}\tau }{2}\nabla \left(\frac{1}{T}\right)\cdot \int \left(f-f_{0}\right)dk+\frac{k_{B}\tau }{2}\int v_{g}\cdot O\left[\nabla {\left(f-f_{0}\right)}^{2}\right]dk\\ =-\frac{\tau }{2}q\cdot \nabla \left(\frac{1}{T}\right)+\frac{k_{B}\tau }{2}\int v_{g}\cdot O\left[\nabla {\left(f-f_{0}\right)}^{2}\right]dk \end{array}$$

Accordingly, the second-order estimation of $s_{f}$ is given by

(13) $$s_{f}=s_{C}-\frac{\tau }{2}q\cdot \nabla \left(\frac{1}{T}\right)+k_{B}\tau \int O\left[\partial_{t}{\left(f-f_{0}\right)}^{2}+v_{g}\cdot \nabla {\left(f-f_{0}\right)}^{2}+\frac{{\left(f-f_{0}\right)}^{3}}{\tau }\right]dk$$

When $\tau \left|\partial_{t}{\left(f-f_{0}\right)}^{2}\right|\ll {\left(f-f_{0}\right)}^{2}$ and $l\left|\nabla {\left(f-f_{0}\right)}^{2}\right|\ll {\left(f-f_{0}\right)}^{2}$ with $l=\left|v_{g}\right|\tau$ denoting the mean free path (MFP), one can obtain a macroscopic approximation as follows

(14a) $$s_{f}≅s_{C}-\frac{\tau }{2}q\cdot \nabla \left(\frac{1}{T}\right)$$

If there exists a well-defined effective thermal conductivity $\lambda_{eff}$, namely, $\nabla T=-{\left(\lambda_{eff}\right)}^{-1}q$, the EIT entropy is formally recovered:

(14b) $$s_{f}≅s_{C}-\frac{\tau }{2\lambda_{eff}T^{2}}q\cdot q$$

In the anisotropic cases, $\lambda_{eff}$ should be replaced by the thermal conductivity tensor $\left[\lambda_{ij}\right]$, and thereupon,

(14c) $$s_{f}≅s_{C}-\frac{\tau }{2T^{2}}q\cdot {\left[\lambda_{ij}\right]}^{-1}\cdot q$$

The remainder term $k_{B}\tau \int O\left[\partial_{t}{\left(f-f_{0}\right)}^{2}+v_{g}\cdot \nabla {\left(f-f_{0}\right)}^{2}\right]dk$ indicates that Equation (14) is restricted to the case with small temporal and spatial derivatives of ${\left(f-f_{0}\right)}^{2}$. It should be pointed out that despite ${\left(f-f_{0}\right)}^{2}\ll \left|f-f_{0}\right|\ll f_{0}$, the derivatives of ${\left(f-f_{0}\right)}^{2}$ can be comparable to or even much larger than the derivative of f.

We now consider $J_{f}$, which is expanded as

(15) $$J_{f}=k_{B}\int v_{g}\left(f-f_{0}\right)\ln\frac{f_{0}+1}{f_{0}}dk+k_{B}\int -\frac{v_{g}{\left(f-f_{0}\right)}^{2}}{2f_{0}\left(f_{0}+1\right)}dk+k_{B}\int v_{g}o{\left(f-f_{0}\right)}^{2}dk$$

The first-order term in the former equation coincides with $J_{C}$ exactly:

(16) $$k_{B}\int v_{g}\left(f-f_{0}\right)\ln\frac{f_{0}+1}{f_{0}}dk=k_{B}\int v_{g}\left(f-f_{0}\right)\frac{\hbar \omega }{k_{B}T}dk=\frac{q}{T}=J_{C}$$

Using the method stated above, the second-order term in Equation (15) can be expressed as

(17) $$k_{B}\int \frac{v_{g}{\left(f-f_{0}\right)}^{2}}{2f_{0}\left(f_{0}+1\right)}dk=-\frac{\tau }{2}Q\cdot \nabla \left(\frac{1}{T}\right)+k_{B}\tau \int v_{g}O\left[\partial_{t}{\left(f-f_{0}\right)}^{2}+v_{g}\cdot \nabla {\left(f-f_{0}\right)}^{2}\right]dk$$

Hence, the entropy-density extra flux is expressed by

(18) $$K=-\frac{\tau }{2}Q\cdot \nabla \left(\frac{1}{T}\right)+k_{B}\tau \int v_{g}O\left[\partial_{t}{\left(f-f_{0}\right)}^{2}+v_{g}\cdot \nabla {\left(f-f_{0}\right)}^{2}+\frac{{\left(f-f_{0}\right)}^{3}}{\tau }\right]dk$$

When $\tau \left|\partial_{t}{\left(f-f_{0}\right)}^{2}\right|\ll {\left(f-f_{0}\right)}^{2}$ and $l\left|\nabla {\left(f-f_{0}\right)}^{2}\right|\ll {\left(f-f_{0}\right)}^{2}$, a second-order approximation emerges: $K≅-\frac{\tau }{2}Q\cdot \nabla \left(\frac{1}{T}\right)$. With well-defined $\lambda_{eff}$, this approximation fulfills the form by Sellitto et al. [1]: $K=\alpha Q\cdot q=\alpha Q^{T}\cdot q$ (Q is symmetrical in phonon heat transport). Besides $s_{f}$ and $J_{f}$, $\sigma_{f}$ can also be expanded:

(19) $$\sigma_{f}=\frac{k_{B}}{\tau }\int \left(f_{0}-f\right)\ln\frac{f_{0}+1}{f_{0}}dk+\frac{k_{B}}{\tau }\int \frac{{\left(f-f_{0}\right)}^{2}}{f_{0}\left(f_{0}+1\right)}dk+\frac{k_{B}}{\tau }\int O{\left(f-f_{0}\right)}^{3}dk$$

Equation (19) implies that $\sigma_{f}$ is a second-order quantity, and combining it with Equation (8) leads to

(20) $$s_{f}=s_{f=f_{0}}-\frac{\tau \sigma_{f}}{2}+k_{B}\int O{\left(f-f_{0}\right)}^{3}dk$$

Consequently, the extra entropic functional $\left(s_{f}-s_{f=f_{0}}\right)$ should be non-positive at least in the near-equilibrium region. Because the remainder term $k_{B}\int O{\left(f-f_{0}\right)}^{3}dk$ does not contain any temporal or spatial derivatives, the approximation $s_{f}≅s_{C}-\frac{\tau \sigma_{f}}{2}$ is more universal than Equation (14). Similar connection between the entropy generation and non-equilibrium contribution to the entropic functional has been derived in Reference [3]. This derivation is based on the decay to equilibrium in the isolated volume element, which does not rely on any specific transport equation. Through this approximation, the entropy balance equation is rewritten as

(21) $$\begin{array}{l} \sigma_{f}=\partial_{t}s_{f}+\nabla \cdot J_{f}≅\partial_{t}\left(s_{f=f_{0}}-\frac{\tau \sigma_{f}}{2}\right)+\nabla \cdot \left(J_{C}+K\right)\\ \Rightarrow \sigma_{f}+\frac{\tau }{2}\partial_{t}\sigma_{f}=\sigma_{C}+\nabla \cdot K \end{array}$$

Equation (21) exhibits a memory behavior between $\sigma_{f}$ and $\left(\sigma_{C}+\nabla \cdot K\right)$, namely,

(22) $$\sigma_{f}\left(x,t\right)=\sigma_{f}\left(x,0\right)+\frac{2}{\tau }\int_{0}^{t}\left(\sigma_{C}+\nabla \cdot K\right)\left(x,t-\xi \right)\exp\left(-\frac{2\xi }{\tau }\right)d\xi$$

Equation (22) does not require small temporal or spatial derivatives, which may be valid in super-transient and large-gradient heat conduction. 

## 3. Fractional Models

In the previous section, we discuss entropic functionals based on the standard BTE, which gives rise to integer-order heat conduction models such as the CV model. There are also fractional-order heat conduction models, i.e., the GCE class [23], whose entropic frameworks have not been studied based on the fractional BTE approach. In the following, the entropic frameworks of the GCE class will be investigated based on fractional BTEs. The first model is $q+\tau_{}^{\gamma }D_{t}^{\gamma }q=-\lambda \nabla T$, which can be derived from a fractional BTE as follows:

(23) $$\tau_{}^{\gamma -1}D_{t}^{\gamma }f+v_{g}\cdot \nabla f=\frac{f_{0}-f}{\tau }$$

where 0<γ≤1 and $D_{t}^{\gamma }$ is the Riemann-Liouville (RL) operator [24]. With the initial value terms neglected, $D_{t}^{1-\gamma }D_{t}^{\gamma }$ equals $\partial_{t}$, and Equation (23) is reformed as

(24) $$\partial_{t}f+\tau_{}^{1-\gamma }D_{t}^{1-\gamma }\left(v_{g}\cdot \nabla f\right)=\tau_{}^{1-\gamma }D_{t}^{1-\gamma }\left(\frac{f_{0}-f}{\tau }\right)$$

Combining Equations (24) and (4) yields

(25) $$\partial_{t}s_{f}=k_{B}\int \left[D_{t}^{1-\gamma }\left(\frac{f_{0}-f}{\tau^{\gamma }}\right)-\tau_{}^{1-\gamma }D_{t}^{1-\gamma }\left(v_{g}\cdot \nabla f\right)\right]\ln\frac{f+1}{f}dk$$

Accordingly, the entropy flux and entropy production rate take the following forms, respectively,

(26) $$\nabla \cdot J_{f}=k_{B}\int \ln\frac{f+1}{f}\tau_{}^{1-\gamma }D_{t}^{1-\gamma }\left(v_{g}\cdot \nabla f\right)dk$$

(27) $$\sigma_{f}=k_{B}\int \ln\frac{f+1}{f}D_{t}^{1-\gamma }\left(\frac{f_{0}-f}{\tau^{\gamma }}\right)dk$$

As the form of $s_{f}$ does not change, the second-order estimation in Equation (13) still holds. Unlike the above case, Equation (26) is an implicit form whose first-order term deviates from $J_{C}$. Due to the existence of fractional operator, $J_{f}$ cannot be expanded directly. Hence, we use $\ln\frac{f+1}{f}=\ln\frac{f_{0}+1}{f_{0}}+O\left(f-f_{0}\right)$, and Equation (26) becomes

(28) $$\begin{array}{l} \nabla \cdot J_{f}=k_{B}\int \left[\ln\frac{f_{0}+1}{f_{0}}+O\left(f-f_{0}\right)\right]\tau_{}^{1-\gamma }D_{t}^{1-\gamma }\left(v_{g}\cdot \nabla f\right)dk\\ =\nabla \cdot \left[\frac{\tau_{}^{1-\gamma }D_{t}^{1-\gamma }q}{T}\right]+\nabla \cdot \int k_{B}v_{g}O\left(f-f_{0}\right)\tau_{}^{1-\gamma }D_{t}^{1-\gamma }fdk\\ \Rightarrow J_{f}=\frac{\tau_{}^{1-\gamma }D_{t}^{1-\gamma }q}{T}+\int k_{B}v_{g}O\left(f-f_{0}\right)\tau_{}^{1-\gamma }D_{t}^{1-\gamma }fdk. \end{array}$$

If f fulfills the condition $\ln\frac{f_{0}+1}{f_{0}}=\frac{\hbar \omega }{k_{B}T}\gg \left|f-f_{0}\right|$, which may be valid in low-temperature situations, a macroscopic approximation arises: $J_{f}≅T^{-1}\tau_{}^{1-\gamma }D_{t}^{1-\gamma }q$. This macroscopic approximation can also be derived from the energy conservation equation. By multiplying Equation (23) by ℏω and integrating it over the wave vector space, we deduce a fractional-order energy balance equation as follows

(29) $$\partial_{t}e=-\tau_{}^{1-\gamma }D_{t}^{1-\gamma }\left(\nabla \cdot q\right)$$

In the presence of near-equilibrium, $\partial_{t}s_{f}≅\partial_{t}s_{f=f_{0}}$ and the entropy balance equation is approximated as

(30) $$\partial_{t}s_{f}≅\partial_{t}s_{f=f_{0}}=\frac{1}{T}\partial_{t}e=-\nabla \cdot J_{\gamma }+\sigma_{\gamma }$$

where $J_{\gamma }=J_{\gamma }\left(x,t\right)$ and $\sigma_{\gamma }=\sigma_{\gamma }\left(x,t\right)$ denote the approximate entropy flux and entropy production rate respectively. Substituting Equation (29) into Equation (30) leads to

(31) $$\partial_{t}s_{f=f_{0}}=\frac{1}{T}\partial_{t}e=-\nabla \cdot \left(\frac{\tau_{}^{1-\gamma }D_{t}^{1-\gamma }q}{T}\right)+\left(\tau_{}^{1-\gamma }D_{t}^{1-\gamma }q\right)\cdot \nabla \left(\frac{1}{T}\right)=-\nabla \cdot J_{\gamma }+\sigma_{\gamma }$$

When γ=1, $J_{\gamma }$ and $\sigma_{\gamma }$ should reduce to the CIT formalism, and therefore, we can derive

(32) $$J_{\gamma }=\frac{\tau_{}^{1-\gamma }D_{t}^{1-\gamma }q}{T}$$

(33) $$\sigma_{\gamma }=\left(\tau_{}^{1-\gamma }D_{t}^{1-\gamma }q\right)\cdot \nabla \left(\frac{1}{T}\right)$$

$J_{\gamma }\left(x,t\right)$ and $\sigma_{\gamma }\left(x,t\right)$ are fundamentally different from the CIT or EIT formalism, which are not determined by instantaneous q but depend on the integrated history of q in [0,t].

The temporal fractional operator can occur in the temperature gradient as well [23]: $q+\tau_{}^{\gamma }D_{t}^{\gamma }q=-\tau_{}^{1-\gamma }D_{t}^{1-\gamma }\left(\lambda \nabla T\right)$, which can be derived from the fractional BTE as follows

(34) $$\tau_{}^{\gamma -1}D_{t}^{\gamma }f+\tau_{}^{1-\gamma }D_{t}^{1-\gamma }\left(v_{g}\cdot \nabla f\right)=\frac{f_{0}-f}{\tau }$$

With the initial value terms neglected, Equation (36) becomes

(35) $$\partial_{t}f+\tau_{}^{2-2\gamma }D_{t}^{2-2\gamma }\left(v_{g}\cdot \nabla f\right)=\tau_{}^{1-\gamma }D_{t}^{1-\gamma }\left(\frac{f_{0}-f}{\tau }\right)$$

The corresponding entropy balance equation is given by

(36) $$s_{f}=k_{B}\int \left[D_{t}^{1-\gamma }\left(\frac{f_{0}-f}{\tau^{\gamma }}\right)-\tau_{}^{2-2\gamma }D_{t}^{2-2\gamma }\left(v_{g}\cdot \nabla f\right)\right]\ln\frac{f+1}{f}dk$$

$\sigma_{f}$ remains Equation (27), while $J_{f}$ fulfills:

(37) $$\nabla \cdot J_{f}=k_{B}\int \ln\frac{f+1}{f}\tau_{}^{2-2\gamma }D_{t}^{2-2\gamma }\left(v_{g}\cdot \nabla f\right)dk$$

Similar to Equation (28), Equation (37) can be expanded as:

(38) $$J_{f}=\frac{\tau_{}^{2-2\gamma }D_{t}^{2-2\gamma }q}{T}+\int k_{B}v_{g}O\left(f-f_{0}\right)\tau_{}^{2-2\gamma }D_{t}^{2-2\gamma }fdk.$$

The energy conservation equation from Equation (34) is given by

(39) $$\partial_{t}e=-\tau_{}^{2-2\gamma }D_{t}^{2-2\gamma }\left(\nabla \cdot q\right)$$

and substituting it into the entropy balance equation leads to

(40) $$\begin{array}{l} \partial_{t}s_{f=f_{0}}=-\nabla \cdot \left(\frac{\tau_{}^{2-2\gamma }D_{t}^{2-2\gamma }q}{T}\right)+\left(\tau_{}^{2-2\gamma }D_{t}^{2-2\gamma }q\right)\cdot \nabla \left(\frac{1}{T}\right)=-\nabla \cdot J_{\gamma }+\sigma_{\gamma }\\ \Rightarrow J_{\gamma }=\frac{\tau_{}^{2-2\gamma }D_{t}^{2-2\gamma }q}{T},\sigma_{\gamma }=\left(\tau_{}^{2-2\gamma }D_{t}^{2-2\gamma }q\right)\cdot \nabla \left(\frac{1}{T}\right) \end{array}$$

Another model is $q+\tau \partial_{t}q=-\tau_{}^{1-\gamma }D_{t}^{1-\gamma }\left(\lambda \nabla T\right)$ [23], which emerges from:

(41) $$\partial_{t}f+\tau_{}^{1-\gamma }D_{t}^{1-\gamma }\left(v_{g}\cdot \nabla f\right)=\frac{f_{0}-f}{\tau }$$

The corresponding entropy balance equation is given by

(42) $$\partial_{t}s_{f}=k_{B}\int \left[\frac{f_{0}-f}{\tau }-\tau_{}^{1-\gamma }D_{t}^{1-\gamma }\left(v_{g}\cdot \nabla f\right)\right]\ln\frac{f+1}{f}dk$$

$\sigma_{f}$ remains Equation (6), while $J_{f}$ is still Equation (25). The last one is a fractional single-phase-lagging (SPL) model [23,25], namely, $q\left(x,t+\tau \right)=-\tau_{}^{1-\gamma }D_{t}^{1-\gamma }\left[\lambda \nabla T\left(x,t\right)\right]$. Discussion on entropic problems for the integer-order SPL model can be found in References [26,27]. The fractional SPL model can emerge from the following BTE

(43) $$D_{t}^{1-\gamma }\left[v_{g}\cdot \nabla f\left(x,t\right)\right]=\frac{f_{0}\left(x,t\right)-f\left(x,t+\tau \right)}{\tau }$$

If a Taylor expansion $f\left(x,t+\tau \right)=f+\tau \partial_{t}f+O\left(\tau^{2}\right)$ is applied to Equation (43), we arrive at $\partial_{t}f+\tau_{}^{1-\gamma }D_{t}^{1-\gamma }\left(v_{g}\cdot \nabla f\right)=\frac{f_{0}-f}{\tau }+O\left(\tau \right)$. The fractional operator occurs in the gradient term and there exists a remainder term O(τ), which is different from Equation (23). When ∇f=0, Equation (23) corresponds to Mittag-Leffler decay to the equilibrium distribution function, which becomes power-law in the long-time limit. For Equation (43), the distribution function must equal to the equilibrium distribution function for any t>τ. Thus, Equation (43) possesses much larger decaying rate than Equation (23). $J_{f}$ remains Equation (25), while $\sigma_{f}$ is given by

(44) $$\sigma_{f}\left(x,t\right)=k_{B}\int \frac{f_{0}\left(x,t\right)-f\left(x,t+\tau \right)+\partial_{t}f\left(x,t\right)}{\tau }\ln\frac{f\left(x,t\right)+1}{f\left(x,t\right)}dk$$

Unlike the above case, the zero-order term of Equation (44) is not zero:

(45) $$\begin{array}{l} \int \frac{f_{0}\left(x,t\right)-f_{0}\left(x,t+\tau \right)+\tau \partial_{t}f_{0}\left(x,t\right)}{\tau }\ln\frac{f_{0}\left(x,t\right)+1}{f_{0}\left(x,t\right)}dk\\ =\frac{1}{T\tau }\left[e\left(x,t\right)+\tau \partial_{t}e\left(x,t\right)-e\left(x,t+\tau \right)\right] \end{array}$$

Therefore, there is only zero-order approximation for $\sigma_{f}$, namely,

(46) $$\sigma_{f}\left(x,t\right)≅\frac{1}{T\tau }\left[e\left(x,t\right)+\tau \partial_{t}e\left(x,t\right)-e\left(x,t+\tau \right)\right]$$

Equation (46) is very different from the above forms, which rely only on the energy density and is independent of the heat flux.

Compte and Metzler have also mentioned entropy framework for fractional transport equations, which will be compared with our results in the following. In the study by Compte and Metzler, the conservation and constitutive equations are independent of each other. A given constitutive equation can be combined with an arbitrary conservation equation. In this work, both conservation and constitutive equations are obtained by the BTE, and their relation is restricted by the BTE as well. A given constitutive equation corresponds to a unique BTE, and hence the conservation equation is not arbitrary. In contrast with the result by Compte and Metzler, the entropic framework of the present paper cannot be separated from the conservation law. Besides the GCE class, there are also more complicated fractional and phonon heat transport equations [28,29,30,31], which deserve further discussion. 

## 4. Conclusions

1. For the BG entropy in phonon heat transport, we provide a second-order approximation, namely, $s_{f}≅s_{C}-\frac{\tau }{2}q\cdot \nabla \left(\frac{1}{T}\right)$, which is valid for both integer-order and fractional-order BTEs. If there exists a well-defined effective thermal conductivity, this approximation will coincide with the EIT entropy. This approximation requires small temporal and spatial derivatives of ${\left(f-f_{0}\right)}^{2}$. We also provide an approximation which does not rely on small temporal and spatial derivatives: $s_{f}≅s_{C}-\frac{\tau \sigma_{f}}{2}$.
2. There are different forms of the entropy flux for different BTEs. For the standard BTE, we obtain the entropy-density extra flux in coincidence with EIT, which is a second-order approximation. In contrast with the standard BTE, the entropy flux for the fractional BTE deviates from the CIT formalism even in the near-equilibrium region. Thus, the form $J=J_{C}+K$ is not applicable for the fractional heat conduction models. Based on the energy conservation equation, we propose a macroscopic form for the entropy flux, namely, $J_{\gamma }=T^{-1}\tau_{}^{F\left(\gamma \right)}D_{t}^{F\left(\gamma \right)}q$, where function F(γ) is determined by the fractional BTE.
3. For the standard BTE, we deduce a convolution form for the entropy production rate, $\sigma_{f}\left(x,t\right)=\sigma_{f}\left(x,0\right)+\frac{2}{\tau }\int_{0}^{t}\left(\sigma_{C}+\nabla \cdot K\right)\left(x,t-\xi \right)\exp\left(-\frac{2\xi }{\tau }\right)d\xi$, which reflects memory or relaxation between $\sigma_{f}$ and $\left(\sigma_{C}+\nabla \cdot K\right)$. Like the entropy flux, the entropy production rate of the fractional BTE can deviate from the CIT formalism in the presence of near-equilibrium. The macroscopic approximation of the entropy production rate usually takes the form $\sigma_{\gamma }=\left[\tau_{}^{F\left(\gamma \right)}D_{t}^{F\left(\gamma \right)}q\right]\cdot \nabla \left(T^{-1}\right)$, while the fractional SPL model corresponds a different expression, $\sigma_{f}\left(x,t\right)≅\frac{1}{T\tau }\left[e\left(x,t\right)+\tau \partial_{t}e\left(x,t\right)-e\left(x,t+\tau \right)\right]$.
4. For fractional models, the entropic functionals perform a history-dependence, which has not been involved in existing phenomenological thermodynamics of irreversible processes [32,33,34,35]. Although our results agree with the framework of EIT in specific cases, Equation (13) indicates possible deviation induced by large temporal and spatial derivatives. In a recent work, Guo et al. [36] investigated the entropic framework for the phonon hydrodynamic model. They observed a deviation from the EIT entropy, which depends on ${\left(\nabla q\right)}_{o}^{S}=\frac{1}{2}\left[\left(\nabla q\right)+{\left(\nabla q\right)}^{T}\right]-\frac{1}{3}\left(\nabla \cdot q\right)I$. Noting that $\nabla \cdot q=-c\partial_{t}T$, the deviation term is then associated with the temporal and spatial derivatives.
5. One possible application scenario in which the non-classical entropic expressions can be important for nanoscale heat transfer is information processing. In essence, it is the entropy transport needed by information erasure that entails heat transfer. Based on conceptual connections between information theory and thermodynamics [37], information erasure can directly correspond to entropy transport, which is commonly achieved through heat transfer. Accordingly, it is necessary to establish the relation between entropy transport and heat transfer, especially when information processing is performed in non-classical cases such as nanoscale.
